# Dual therapy with darunavir/r plus etravirine is safe and effective as switching therapy in antiretroviral experienced HIV-patients. The BITER Study

**DOI:** 10.7448/IAS.17.4.19803

**Published:** 2014-11-02

**Authors:** Joaquín Portilla, Piedad Arazo, Josefa Crusells, Laura Pérez-Martínez, Onofre Martínez-Madrid, Vicente Boix, Javier Moreno, Vicente Navarro, Teresa Rubio, Sergio Reus, Carlos Galera, Enrique Bernal, Francisco Jover, Concepcion Amador, David Baño, Esperanza Merino, Pablo Saiz-de-la-Hoya

**Affiliations:** 1 Unidad Enfermedades Infecciosas, Hospital General Universitario de Alicante, Alicante, Spain; 2 Unidad Enfermedades Infecciosas, Hospital Universitario Miguel Servert, Zaragoza, Spain; 3 Servicio Enfermedades Infecciosas, Hospital Clínico Universitario Lozano Blesa, Zaragoza, Spain; 4 Servicio Enfermedades Infecciosas, Hospital de San Pedro, La Rioja, Spain; 5 Unidad Enfermedades Infecciosas, Hospital Gral Universitario Santa Lucía, Cartagena, Spain; 6 Unidad Enfermedades Infecciosas, Hospital General Universitario Alicante, Alicante, Spain; 7 Unidad Enfermedades Infecciosas, Hospital Elche-Vinalopó, Elche, Spain; 8 Unidad Enfermedades Infecciosas, Hospital Reina Sofia de Navarra, Tudela, Spain; 9 Unidad VIH, Hospital General Universitario Virgen Arrixaca, Murcia, Spain; 10 Unidad Enfermedades Infecciosas, Hospital Universitario Reina Sofia de Murcia, Murcia, Spain; 11 Unidad Enfermedades Infecciosas, Hospital Universitario San Juan, Alicante, Spain; 12 Unidad Enfermedades Infecciosas, Hospital Marina Baixa, Villajoyosa, Spain; 13 Unidad VIH, Hospital de Torrevieja, Torrevieja, Spain; 14 Servicios Médicos Penitenciarios, Centro Penitenciario Alicante 1, Alicante, Spain

## Abstract

**Introduction:**

Switching therapy studies are usually designed as second-line antiretroviral treatment (ART) in patients without previous virologic failures. Combined ART (cART) with DRV/r and ETR has a good pharmacokinetic profile, high genetic barrier and has been proved as rescue therapy. The aim of our study was to analyze efficacy and safety of therapy with DRV/r plus ETR in treatment experienced HIV-patients with previous therapeutic failures that need to switch ART. We present results at first 24 weeks.

**Methods:**

Multicentre retrospective observational study. Inclusion criteria: adult HIV-patients on ART with HIV-VL <1000 cop/mL who started their ART with DRV/r (600/100 bid or 800/100 qd)+ETR by adverse events, non-adherence, tolerability or prevention of future complications. Patients with acute AIDS events, HBV, pregnancy, drug addiction or previous selected mutations to DRV or ETR were excluded.

**Results:**

Ninety-nine patients were included, mean age: 47 years (r: 22–79); 70% men, 40.4% previous AIDS event and 39.3% HCV. Ninety-one patients had received ≥3 cART regimens and 45≥5, 75 patients had HIV-VL <50 cop/mL and 24 low-level viremia (LLV): 297.5±261.4 cop/mL, CD4+ 568±279 cells/µL. ART before switching: NRTI+PI/r (33%), NNRTI (17%), PI/r+NNRTI (23%), PI/r+INI (13%), other (14%). Main reason to switching was: toxicity/intolerance 50 patients (renal 32%, gastrointestinal: 14%, hyperlipidaemia 10%; osteopenia/osteoporosis: 6%); improving adherence 26 patients; prevention of complications 19 patients. Nine subjects withdrew ART during follow-up because: intolerance or new toxicity three; non-adherence two; simplification to DRV/r monotherapy two; persistence of previous toxicity one; virologic failure one. At week 24, among patients who continued with DRV/r+ETR (n=90): 81 (89%) had VL<50 cop/mL, in those with with HIV-VL<50 at baseline (67/90), 94% persisted with <50 cop., and in those with LLV (24/90), 61% (n=14) achieved a VL<50 cop. We didn't observe any significant difference in lab parameters between baseline and week 24. Estimated glomerular filtrate rate increased from 83.4±24.7 to 88.5±56.8 mL/min, p=NS. Regarding reason to switching, it improved in 42 cases, no changes: 20 cases; worsened: 4 cases, and non-applicable or unknown: 24 cases.

**Conclusions:**

Switching to dual therapy with DRV/r+ETR is an effective strategy in selected heavily experienced ART patients, even in those with LLV (<1000 cop/mL). This cART is safe and well tolerated, can reduce number of pills and improve adherence.

